# The role of β-arrestin2-dependent signaling in thoracic aortic aneurysm formation in a murine model of Marfan syndrome

**DOI:** 10.1152/ajpheart.00291.2015

**Published:** 2015-09-14

**Authors:** James W. Wisler, Emily M. Harris, Michael Raisch, Lan Mao, Jihee Kim, Howard A. Rockman, Robert J. Lefkowitz

**Affiliations:** ^1^Department of Medicine, Duke University Medical Center, Durham, North Carolina;; ^2^Department of Dermatology, Duke University Medical Center, Durham, North Carolina;; ^3^Department of Cell Biology, Duke University Medical Center, Durham, North Carolina;; ^4^Department of Molecular Genetics, Duke University Medical Center, Durham, North Carolina;; ^5^Department of Biochemistry, Duke University Medical Center, Durham, North Carolina; and; ^6^Howard Hughes Medical Institute, Duke University Medical Center, Durham, North Carolina

**Keywords:** Marfan syndrome, thoracic aortic aneurysm, angiotensin 1a receptor, β-arrestin2

## Abstract

Ang II type 1a receptor (AT_1a_R)-mediated activation of MAPKs contributes to thoracic aortic aneurysm (TAA) development in Marfan syndrome (MFS). β-Arrestin2 (βarr2) is known to mediate AT_1a_R-dependent MAPK activation, as well as proproliferative and profibrotic signaling in aortic vascular smooth muscle cells. Therefore, we investigated whether βarr2-dependent signaling contributes to TAA formation in MFS. We used a murine model of MFS [fibrillin (*Fbn*)^C1039G/+^] to generate an MFS murine model in combination with genetic βarr2 deletion (*Fbn*^C1039G/+^/*βarr2*^−/−^). *Fbn*^C1039G/+^/*βarr2*^−/−^ mice displayed delayed aortic root dilation compared with *Fbn*^C1039G/+^ mice. The mRNA and protein expression of several mediators of TAA formation, including matrix metalloproteinase (MMP)-2 and -9, was reduced in the aorta of *Fbn*^C1039G/+^/*βarr2*^−/−^ mice relative to *Fbn*^C1039G/+^ mice. Activation of ERK1/2 was also decreased in the aortas of *Fbn*^C1039G/+^/*βarr2*^−/−^ mice compared with *Fbn*^C1039G/+^ animals. Small interfering RNA targeting βarr2 inhibited angiotensin-stimulated expression of proaneurysmal signaling mediators in primary aortic root smooth muscle cells. Angiotensin-stimulated expression of the proaneurysmal signaling mediators MMP-2 and -9 was inhibited by blockade of ERK1/2 or the EGF receptor, whereas blockade of the transforming growth factor-β receptor had no effect. These results suggest that βarr2 contributes to TAA formation in MFS by regulating ERK1/2-dependent expression of proaneurysmal genes and proteins downstream of the AT_1a_R. Importantly, this demonstration of the unique signaling mechanism by which βarr2 contributes to aneurysm formation identifies multiple novel, potential therapeutic targets in MFS.

## Abstract (precis)

*This manuscript demonstrates that β-arrestin2 mediates thoracic aortic aneurysm formation in a murine model of Marfan syndrome (MFS) by regulating proaneurysmal signaling. This work identifies a novel signaling cascade that contributes to aortic aneurysm formation as well as several potential, previously unappreciated therapeutic targets in MFS*.

## NEW & NOTEWORTHY

*This manuscript demonstrates that β-arrestin2 mediates thoracic aortic aneurysm formation in a murine model of Marfan syndrome (MFS) by regulating proaneurysmal signaling. This work identifies a novel signaling cascade that contributes to aortic aneurysm formation as well as several potential, previously unappreciated therapeutic targets in MFS*.

marfan syndrome (MFS) is a systemic, autosomal dominant disease of connective tissue cause by mutations in the gene encoding fibrillin-1, a major component of the microfibril network of the ECM (13, 15). This disorder is characterized by a variety of clinical manifestations, including elongated limbs, bony overgrowth, craniofacial abnormalities, subluxation of the lens of the eye, as well as myxomatous degeneration of the cardiac valves, particularly the mitral valve. The leading cause of death in MFS is secondary to complications related to the development of thoracic aortic aneurysms (TAAs) (6).

Work over the past two decades has demonstrated that TAA formation in MFS results from multiple dysregulated signaling pathways involving a complex network of signaling mediators and receptors. However, many of these pathways are poorly understood with regard to their contribution to TAA development. Consequently, an enhanced understanding of the pathogenic signaling mechanisms involved in TAA formation may stimulate the development of more efficacious, safe, or targeted therapeutic options to prevent or treat this deadly complication of MFS.

In addition to aberrant transforming growth factor-β (TGF-β) signaling, Habashi et al. (19) identified that blockade of the Ang II (ANG) type 1a receptor (AT_1a_R) inhibited TAA development in a murine model of MFS. The beneficial effect of AT_1a_R blockade on aortic root dilation was independent of the hemodynamic effects of losartan, and identified AT_1a_R-mediated signaling was pathogenic in MFS (19). It was subsequently shown that in addition to activation of TGF-β ligands and receptors, AT_1a_R-mediated signaling contributed to TAA development via upregulation of proaneurysmal signaling mediators, such as matrix metalloproteinases (MMPs) (25).

The AT_1a_R represents a prototypical G protein-coupled receptor (GPCR). The canonical signal transduction paradigm of GPCRs is characterized by an agonist-induced conformational change upon ligand binding to the receptor. This conformational change allows the receptor to interact with G proteins, as well as the non-G protein transducers, β-arrestins (βarr) 1 and 2 (11). Originally identified as proteins that mediate receptor desensitization (4, 30) and internalization (17, 23, 24), work over the past two decades has demonstrated that arrestins also mediate G protein-independent signaling for multiple GPCR types [for review, see DeWire et al. (11)]. βarr2 has been shown to mediate AT_1a_R signaling, including activation of MAPKs, such as ERK1/2, by serving as a scaffold for proteins that facilitate signaling to ERK1/2 (26). AT_1a_R-mediated, βarr2-dependent ERK1/2 activation is both spatially and temporally distinct from ERK1/2 activated via G_q_ proteins (3). This distinct signaling profile, engendered by βarrs, leads to unique physiologic (22) and pharmacologic (34) properties in both receptor- and tissue-specific manners.

Signaling within vascular smooth muscle cells (VSMCs) is thought to play a key role in the pathogenesis of TAA in MFS (5, 29). In VSMCs, ANG signaling via the AT_1a_R stimulates several cellular processes known to be involved in aneurysm formation, including cellular proliferation, migration, fibrosis, and protein synthesis (12, 22, 31, 35). SMAD2 and -3 (31, 35) as well as ERK1/2 (21) have been shown to mediate these cellular processes independent of one another. Aberrant signaling within VSMCs, including ERK signaling, has been linked to TAA development in MFS (18). βarr2 has also been shown to have a role in AT_1a_R-mediated cellular proliferation, migration, fibrosis, and protein synthesis, independent of G protein activation (12, 22, 33). Recent work has also identified βarr2 as a necessary signaling mediator for ANG-induced expression of several proaneurysmal mediators, including MMP-2 and -9, in the murine aorta (33).

MMP-2 and -9 have previously been identified as playing a key role in the pathogenesis of TAA formation in MFS via enhanced elastolysis of the elastic matrix with resultant impaired structural integrity of the aortic wall (8, 9, 36). Production of MMP-2 and -9 has been shown to be both AT_1a_R (33, 38) and ERK dependent (14, 28) in aortic VSMCs and as noted above, is βarr2 dependent in the murine abdominal aorta (33). In addition, upregulation of MMPs, including MMP-2 and -9, has been identified in VSMCs from patients with MFS (29), as well as VSMCs in a murine model of MFS (5). Therefore, we hypothesized that AT_1a_R-mediated, βarr2-dependent signaling would mediate TAA formation in MFS by regulating the expression of the proaneurysmal signaling mediators MMP-2 and -9 in the murine aorta.

Here, we examined the role of βarr2-dependent signaling in TAA formation in MFS. We demonstrate that *Fbn*^C1039G/+^/βarr2 deletion (*βarr2*^−/−^) mice display delayed aortic root dilation compared with *Fbn*^C1039G/+^ mice. Activation of ERK1/2, but not SMAD2/3, is reduced in the aortic root of *Fbn*^C1039G/+^/*βarr2*^−/−^ mice compared with *Fbn*^C1039G/+^ mice, as assessed by Western blot analysis. By both quantitative RT-PCR (qRT-PCR) and Western blot, we demonstrate that MMP-2 and -9 gene and protein expression is reduced in *Fbn*^C1039G/+^/*βarr2*^−/−^ mice compared with *Fbn*^C1039G/+^ mice. We demonstrate directly in primary aortic root VSMCs that βarr2 modulates AT_1a_R-mediated MMP-2 and -9 expression. We also show that this AT_1a_R-mediated, βarr2-dependent proaneurysmal signaling pathway is dependent on ERK activation and transactivation of the EGF receptor (EGFR) and is independent of TGF-β signaling.

## MATERIALS AND METHODS

### 

#### Study design.

The objective of this work was to evaluate whether βarr2-dependent signaling contributes to TAA formation in MFS. This project was carried out using a murine model of MFS, as well as tissue harvested from these animals and primary aortic root VSMCs harvested from Sprague-Dawley rats. For in vivo study, a power calculation was performed a priori to determine the necessary sample size. To detect a 10% difference in aortic root diameter with an α value of 0.05, a β value of 0.20, and a σ value of 0.15, 13 animals were required for each group. Transthoracic echocardiography was planned to continue until 36 wk of age, after which time, tissue would be harvested for analysis. Data were collected and analyzed in an intention-to-treat format with the primary outcome measure specified as aortic root diameter, as assessed by transthoracic echocardiography. As noted below, ECG interpreters were blinded to genotype.

#### Materials.

ANG was synthesized by GenScript USA (Piscataway, NJ) to >98% purity; quality control was by HPLC and mass spectrometry. For stimulation of primary cells, the concentration of ANG was 100 nM. Losartan (final concentration of 1 μM) was purchased from Sigma-Aldrich (St. Louis, MO). The MEK1/2 inhibitor U0126 (final concentration of 5 μM) and EGFR-specific inhibitor tyrphostin AG1478 (final concentration of 250 nM) were purchased from Calbiochem (San Diego, CA). Erlotinib (final concentration of 5 mg/ml) was purchased from Selleck Chemicals (Houston, TX). TGF-β neutralizing antibody was purchased from R&D Systems (Minneapolis, MN).

#### Synthesis of small interfering RNAs.

Small interfering RNAs (siRNAs) were synthesized as described previously (21). The siRNA sequences targeting βarr2 are 5′-GGACCGCAAAGUGUUUGUG-3′ and 5′-CCAACCTCATTGAATTCGA-3′, corresponding to the positions 150–168 and 1,115-1,133 relative to the start codon (2). A nonsilencing RNA duplex (5′-AAUUCUCCGAACGUGUCACGU-3′), as stipulated by the manufacturer, was used as a control.

#### Cell culture.

Primary aortic root VSMCs were harvested from 150 g male Sprague-Dawley rats in the following manner: the thoracic aorta was excised from the aortic root to the brachiocephalic artery. Individual aortic roots were kept in separate dishes, and VSMCs were obtained by enzymatic digestion, as described previously (22), and maintained in DMEM, supplemented with 10% FBS and 1% penicillin-streptomycin. Slow growing, early passage cells (<5) were used for assay. Cultured cells from individual animals were kept separate throughout culturing and experimentation.

#### Experimental animals.

*Fbn*^C1039G/+^ mice (C57BL/6J background) were crossed with *βarr2*^−/−^ mice (C57BL/6J background) to generate *Fbn*^C1039G/+^/*βarr2*^−/−^ animals. Littermate controls were used for in vivo study. Research with animals carried out for this study was handled according to approved protocols and animal welfare regulations of Duke University Medical Center's Institutional Review Boards. Male Sprague-Dawley animals (150 g) were purchased from The Jackson Laboratory (Bar Harbor, ME).

#### Transthoracic echocardiography.

Transthoracic two-dimensional echocardiography was performed using a Vevo 2100 echocardiograph (FUJIFILM VisualSonics, Toronto, ON, Canada). Images were analyzed using Vevo 2100 software (FUJIFILM VisualSonics). Immediately before imaging, animals were anesthetized with a subcutaneous injection of ketamine/xylazine (90 and 4.5 mg/kg, respectively). Serial ECGs consisted of apical and short- and long-axis images of the heart, as well as aortic root and ascending aorta, and were obtained beginning at 8 wk and then every 4 wk thereafter until 36 wk of age. Two independent readers, blinded to animal genotype, measured aortic root diameter on three different images at each time point for each animal. Average aortic root diameter was calculated, and a third independent reader, also blinded to animal genotype, read any image for which the difference between readers 1 and 2 fell outside of 1 SD of the average difference between readers 1 and 2. When a third read was obtained, all three measurements were averaged to determine the aortic root diameter.

#### Hemodynamic study.

After anesthetizing the animal with a subcutaneous injection of ketamine/xylazine (90 and 4.5 mg/kg, respectively), a high-fidelity transducer was inserted into the right carotid artery. Blood pressure was recorded continuously with a pressure-recording system (ADInstruments, Houston, TX). Continuous pressures were recorded in the proximal aorta, as well as in the left ventricle.

#### Erlotinib infusion.

Beginning at 8 wk of age, transthoracic ECG was obtained as above. After anesthetizing the animal with subcutaneous injection of ketamine/xylazine (90 and 4.5 mg/kg, respectively), a subcutaneous osmotic pump (Alzet 2004; Durect, Cupertino, CA), containing either vehicle or erlotinib (5 mg/ml), was placed in the subcutaneous tissue immediately lateral to the spine on the posterior of the animal. After 4 wk, the initially placed pump was removed and replaced with a second pump containing the same treatment (vehicle vs. erlotinib). A follow-up transthoracic ECG was obtained after 8 wk of treatment to assess aortic root diameter, as detailed above.

#### Immunoblotting.

Immunoblotting was performed using a modification of the methods of Kim et al. (21). For immunoblotting of stimulated VSMCs, 80–90% confluent VSMCs in six-well plates were starved in serum-free medium for at least 48 h. Cells were stimulated for 15 min [for phosphorylated (p)ERK1/2 and pSMAD2/3 detection] or 24 h (for MMP-2 and -9 and pSMAD2/3). After stimulation, directly adding 2× SDS-sample buffer, followed by sonication with a microtip at 30% amplitude for 15 s, solubilized monolayers. Equal amounts of cellular extracts were separated on 4–20% Tris-glycine polyacrylamide gels and transferred onto nitrocellulose membranes for immunoblotting.

**Fig. 1.**
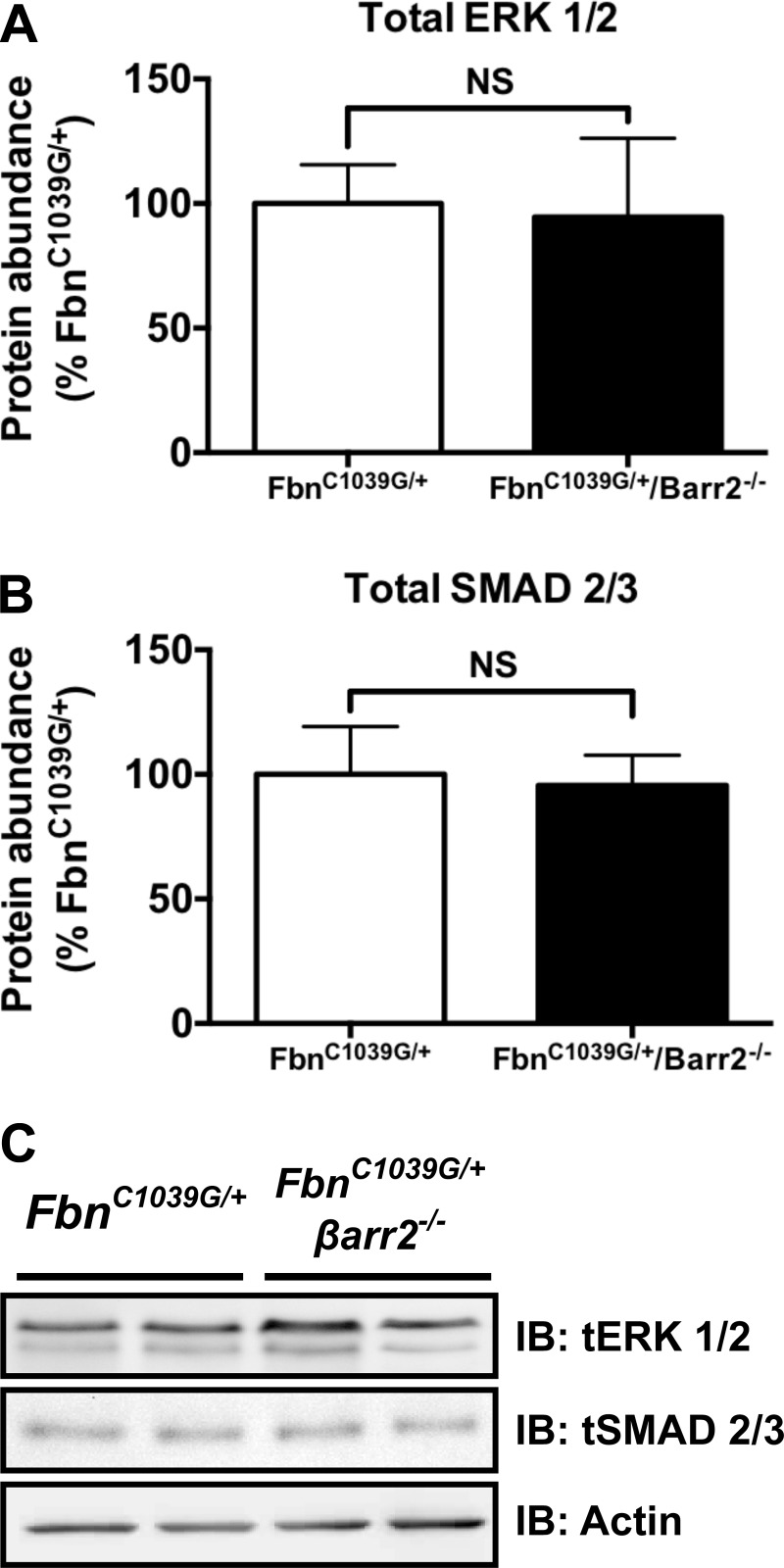
Protein expression of total ERK1/2 and total SMAD2/3 is equivalent in thoracic aortic aneurysm (TAA) tissue from fibrillin (*Fbn*)^C1039G/+^/β-arrestin2 deletion (*βarr2*^−/−^) mice relative to *Fbn*^C1039G/+^ mice. *A*: total ERK1/2 levels do not differ significantly between *Fbn*^C1039G/+^ and *Fbn*^C1039G/+^/*βarr2*^−/−^ (Barr2^−/−^) mice. NS, nonsignificant. *B*: total SMAD2/3 levels do not differ significantly between *Fbn*^C1039G/+^ and *Fbn*^C1039G/+^/*βarr2*^−/−^ mice. *C*: Western blot [immunoblot (IB)] of total (t) ERK1/2 and total SMAD2/3 in TAA tissue samples from *Fbn*^C1039G/+^ and *Fbn*^C1039G/+^/*βarr2*^−/−^. Samples were obtained between 12 and 16 wk of age; *Fbn*^C1039G/+^ (*n* = 5) and *Fbn*^C1039G/+^/*βarr2*^−/−^ (*n* = 4).

For immunoblotting of aortic tissue, thoracic aortas were obtained, extending from the aortic root to immediately proximal to the left subclavian artery, from animals aged 12–16 wk. Aortic tissue was ground using a mechanical tissue homogenizer and lysed in a buffer containing 50 mM Tris-Cl, pH 7.4, 1 mM EDTA, 1% Nonidet P-40, 0.1% SDS, 150 mM NaCl, 0.5% sodium deoxycholate, and Halt protease and phosphatase inhibitor cocktail (Pierce Biotechnology, Rockford, IL). Rotating for 3 h at 4°C solubilized the lysate. Insoluble material was separated by centrifugation at 10,000 rpm for 15 min. Protein concentration of the cleared lysate was determined by the bicinchoninic acid protein assay kit (Pierce Biotechnology). Equal amounts of lysate were mixed with 2× SDS-sample buffer, and 20 mg of protein was loaded onto 4–20% Tris-glycine polyacrylamide gels with Western blot performed as above. pERK1/2, pSMAD2/3, and MMP-2 and -9 were detected by immunoblotting with rabbit polyclonal anti-pp44/42 MAPK (1:2,000; Cell Signaling Technology, Danvers, MA), anti-pSMAD2/3 (1:2,000; Santa Cruz Biotechnology, Dallas, TX), anti-MMP-2 (1:100; Santa Cruz Biotechnology), and anti-MMP-9 (1:100; Santa Cruz Biotechnology). Horseradish peroxidase-conjugated secondary antibodies (1:2,000) were purchased from Amersham Biosciences (Piscataway, NJ). Chemiluminescent detection was performed using the SuperSignal West Pico and Femto reagents (Pierce Biotechnology), and all immunoblots were imaged and quantified using the bio-imaging system (Syngene USA, Frederick, MD). Equal loading was determined using an anti-β-actin antibody (1:5,000; Sigma-Aldrich), and levels of pERK, pSMAD2/3, and MMP-2 and -9 were normalized to the loading control. To ensure validity of anti-β-actin as a loading control, we confirmed that no change in the levels of total ERK1/2 (1:5,000; Cell Signaling Technology) or total SMAD2/3 (1:200; Santa Cruz Biotechnology) was present between *Fbn*^C1039G/+^ and *Fbn*^C1039G/+^/*βarr2*^−/−^ mice (Fig. 1).

#### Quantitative RT-PCR.

For qRT-PCR of stimulated VSMCs, 80–90% confluent VSMCs in six-well plates were starved in serum-free medium for at least 12 h. After stimulation (18–24 h), monolayers were washed in ice-cold PBS and RNA extracted using an RNeasy Mini Kit (Qiagen USA), according to the manufacturer's instructions. For qRT-PCR of aortic tissue, mouse aortas were extracted as noted above. Tissue was ground using a mechanical tissue homogenizer and solubilized in lysis buffer provided in the RNeasy Mini Kit (Qiagen USA). RNA was then extracted, according to the manufacturer's protocol. RNA quantity was determined using a NanoDrop 2000 spectrophotometer (Thermo Scientific, Wilmington, DE). RNA (50 ng) was used for subsequent reverse transcription and cDNA synthesis. Genomic DNA was eliminated, and reverse transcription was performed using an RT^2^ First Strand cDNA Synthesis Kit (Qiagen USA), according to the manufacturer's protocol. qRT-PCR arrays were performed using the RT^2^ Profiler PCR fibrosis arrays (Qiagen USA), according to the manufacturer's instructions for either mouse or rat species where appropriate. qRT-PCR was also performed using primers for selected genes, including MMP-2, MMP-9, cyclooxygenase-2, monocyte chemoattractant protein-1, and collagen 1a1 (col 1a1; Sigma-Aldrich) and TGF-β1, thrombospondin, and SMAD2 (RealTimePrimers.com, Elkins Park, PA; Table 1). qRT-PCR was performed on an ABI StepOnePlus Real-Time PCR System (Thermo Fisher Scientific, Grand Island, NY) and used SYBR Green/ROX qRT-PCR Mastermix (Qiagen USA).

**Table 1.** Quantitative RT-PCR primer sequences

Target Gene, Mouse	Sequence, 5′ → 3′
β-Actin	For: AAGAGCTATGAGCTGCCTGA
	Rev: TACGGATGTCAACGTCACAC
SMAD2	For: GGAACCTGCATTCTGGTGTT
	Rev: ACGTTGGAGAGCAAGCCTAA
Cyclooxygenase 2	For: TGATCGAAGACTACGTGCAA
	Rev: GTGAGTCCATGTTCCAGGAG
Matrix metalloproteinase 2	For: CACCTGGTTTCACCCTTTCT
	Rev: GAGGAGGGGAACCATCACTA
Matrix metalloproteinase 9	For: CTCACTCACTGTGGTTGCTG
	Rev: TGGTTATCCTTCCTGGATCA
Monocyte chemoattractant protein 1	For: TGCTACTCATTCACCAGCAA
	Rev: GTCTGGACCCATTCCTTCTT
Collagen type 1a1	For: CCAGCTGACCTTCCTGCGCC
	Rev: GGAGAGGCCCCAAGAGGGGG
Transforming growth factor-β1	For: GCTACCATGCCAACTTCTGT
	Rev: CGTAGTAGACGATGGGCAGT
Thrombospondin 1	For: AAAGCCAAAGCGCCTATTTA
	Rev: TGGCGGTGAGTTCTAGTGAG
Target Gene, Mouse	Sequence, 5′ → 3′
β-Actin	For: CACACTGTGCCCATCTATGA
	Rev: CCGATAGTGATGACCTGACC
SMAD2	For: GGAACCTGCATTCTGGTGTT
	Rev: ACGTTGGAGAGCAAGCCTAA
Cyclooxygenase 2	For: TGTACCCGGACTGGATTCTA
	Rev: TAAGTTGGTGGGCTGTCAAT
Matrix metalloproteinase 2	For: GGAGCGACGTAACTCCACTA
	Rev: AAGTGAGAATCTCCCCCAAC
Matrix metalloproteinase 9	For: ACTTCTGGCGTGTGAGTTTC
	Rev: TGTATCCGGCAAACTAGCTC
Monocyte chemoattractant protein 1	For: TTGTCACCAAGCTCAAGAGA
	Rev: GGTTGTGGAAAAGAGAGTGG
Collagen type 1a1	For: CCAGCTGACCTTCCTGCGCC
	Rev: GGAGAGGCCCCAAGAGGGGG
Transforming growth factor-β1	For: CGGACTACTACGCCAAAGAA
	Rev: TTGCTCCACAGTTGACTTGA
Thrombospondin 1	For: AAAGCCAAAGCGCCTATTTA
	Rev: TGGCGGTGAGTTCTAGTGAG

For, forward; Rev, reverse.

#### siRNA transfection.

Immediately before transfection, 80–90% confluent primary VSMCs plated in 100 mm dishes were placed in DMEM, containing 10% FBS without antibiotics. DharmaFECT 2 Transfection Reagent (GE Dharmacon, Lafayette, CO) was diluted in serum-free medium (0.1/100 μl medium) and allowed to equilibrate for 5 min. Meanwhile, siRNA was diluted to 50 nM in serum-free medium and also allowed to equilibrate for 5 min, according to the manufacturer's instructions. The tube containing diluted siRNA was then added drop-wise to the tube containing the transfection reagent and was mixed vigorously and allowed to equilibrate at room temperature for 20 min. The transfection reaction was then added drop-wise to 100 mm cell plates. After 48–60 h, cells were split into six-well dishes with antibiotic-free media. Twenty-four hours after splitting, the media were removed and replaced with serum-free media containing 0.1% BSA. Cells were serum starved for 48 h and stimulated as above. Immunoblotting was carried out as described above.

#### Statistical analysis.

Data are expressed as means ± SE. Statistical analysis was performed using Student's paired *t*-test, one-way ANOVA with Bonferroni multiple comparisons post hoc testing, two-way ANOVA, or repeated-measures two-way ANOVA with post hoc testing (GraphPad Prism; GraphPad Software, La Jolla, CA) where appropriate. *P* < 0.05 was considered statistically significant. Normality of the data was confirmed by D'Agostino-Pearson omnibus test or Shapiro-Wilk test, where appropriate. Outliers were identified and excluded by use of Grubbs' test.

## RESULTS

### 

#### βarr2 contributes to TAA development in a murine model of MFS.

**Fig. 2.**
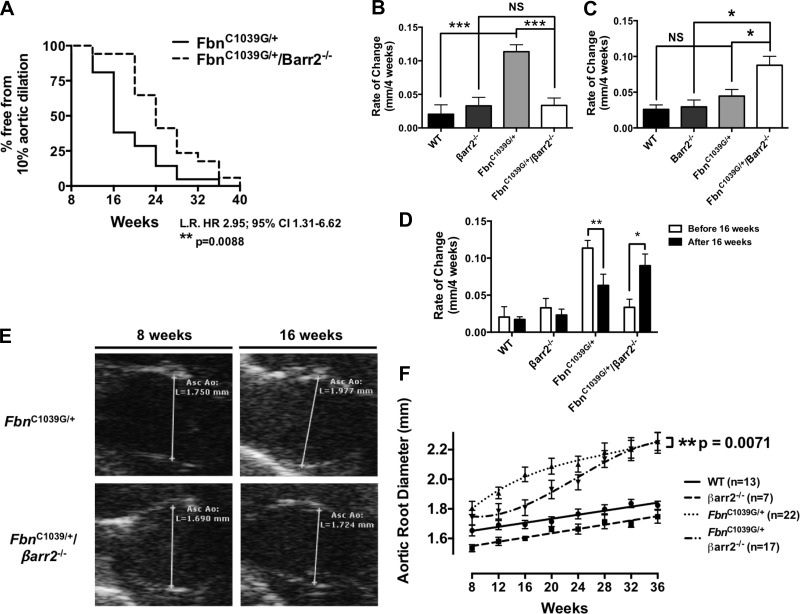
βarr2 contributes to TAA formation in a murine model of Marfan syndrome (MFS). *A*: the median time to 10% aortic dilation is delayed in *Fbn*^C1039G/+^/*βarr2*^−/−^ mice compared with *Fbn*^C1039G/+^ mice [median time to onset, 16 vs. 24 wk; hazard ratio (HR), 2.95; 95% confidence interval (CI), 1.31–6.62; **P = 0.0088 by log-rank test (LR)]. *B*: before 16 wk of age, the rate of aortic root dilation is less in *Fbn*^C1039G/+^/*βarr2*^−/−^ mice relative to *Fbn*^C1039G/+^ mice and similar to *βarr2*^−/−^ mice (***P < 0.001). WT, wild-type; NS, nonsignificant. *C*: after 16 wk, the rate of aortic root dilation is significantly larger in *Fbn*^C1039G/+^/*βarr2*^−/−^ mice relative to *Fbn*^C1039G/+^ mice (*P < 0.05). *D*: the rate of aortic root dilation slows over time in *Fbn*^C1039G/+^ mice (**P < 0.01), whereas the rate of aortic root dilation increases over time in *Fbn*^C1039G/+^/*βarr2*^−/−^ mice (*P < 0.05). *E*: by 16 wk of age, aortic root diameter is smaller in *Fbn*^C1039G/+^/*βarr2*^−/−^ mice compared with *Fbn*^C1039G/+^ mice (1.81 ± 0.05 vs. 2.03 ± 0.05 mm; *P* < 0.05, repeated-measures, 2-way ANOVA with Bonferroni post-testing). Asc Ao, ascending aorta. *F*: *Fbn*^C1039G/+^/*βarr2*^−/−^ mice display delayed aortic root dilation compared with *Fbn*^C1039G/+^ mice (**P = 0.0071 by repeated-measures, 2-way ANOVA). WT, *n* = 13; *βarr2*^−/−^, *n* = 7; *Fbn*^C1039G/+^, *n* = 22; *Fbn*^C1039G/+^/*βarr2*^−/−^, *n* = 17.

To assess directly the role of βarr2 in TAA development in MFS, we generated *Fbn*^C1039G/+^/*βarr2*^−/−^ mice. There were no differences in aortic blood pressure between any genotypes, suggesting that genetic deletion of *βarr2* has no significant hemodynamic effects (Table 2). With the use of transthoracic echocardiography, we observed significantly delayed aortic root dilation in *Fbn*^C1039G/+^/*βarr2*^−/−^ mice relative to *Fbn*^C1039G/+^ mice. The time to achieve aortic dilation of 10% over baseline was markedly prolonged in *Fbn*^C1039G/+^/*βarr2*^−/−^ relative to *Fbn*^C1039G/+^ mice (median time to onset, 24 vs. 16 wk; hazard ratio, 2.95; 95% confidence interval, 1.31–6.62; *P* = 0.0088; Fig. 2*A*).

**Table 2.** Hemodynamic measurements of study animals

	WT	*βarr2*^−/−^	*Fbn^C1039G/^*^+^	*Fbn^C1039G/^*^+^/β*arr2^−/−^*
Heart rate, beats/min	396.5 ± 20.6	402.4 ± 21.0	389.9 ± 19.8	410.1 ± 8.5
Systolic blood pressure, mmHg	141.0 ± 8.5	134.4 ± 8.7	130.3 ± 7.9	132.2 ± 7.0
Diastolic blood pressure, mmHg	99.0 ± 3.5	94.4 ± 5.4	81.8 ± 7.0	85.9 ± 5.3

No significant differences were observed in the heart rates, systolic blood pressures, or diastolic blood pressures between wild-type (WT) and fibrillin (*Fbn*)^C1039G/+^ mice, between *Fbn*^C1039G/+^ and *Fbn*^C1039G/+^/β-arrestin2 deletion (*βarr2*^−/−^) mice, or between *βarr2* ^−/−^ and *Fbn*^C1039G/+^/*βarr2* ^−/−^ mice. Data are expressed as means ± SE; *n* = 12 for WT, *n* = 7 for *βarr2* ^−/−^, *n* = 20 for *Fbn*^C1039G/+^, and *n* = 15 for *Fbn*^C1039G/+^/*βarr2*^−/−^.

Over time, the difference in aortic root diameter between *Fbn*^C1039G/+^ and *Fbn*^C1039G/+^/*βarr2*^−/−^ mice decreased and approached similar values by 28 wk of age. This appears to be the result of two factors: *1*) the rate of aortic root dilation slows over time in *Fbn*^C1039G/+^ mice, and *2*) the rate of aortic root dilation increases over time in *Fbn*^C1039G/+^/*βarr2*^−/−^ mice (Fig. 2, *B–D*). From age 8 to 16 wk, the rate of change in aortic root diameter in *Fbn*^C1039G/+^/*βarr2*^−/−^ mice was significantly lower than that of *Fbn*^C1039G/+^ mice (0.03 ± 0.01 vs. 0.11 ± 0.01 mm/4 wk; *P* < 0.001) and not significantly different from that of *βarr2*^−/−^ mice [0.03 ± 0.01 vs. 0.03 ± 0.01 mm/4 wk; *P* = nonsignificant (NS); Fig. 2*B*]. Beyond 16 wk of age, however, the rate of change in aortic root diameter in *Fbn*^C1039G/+^/*βarr2*^−/−^ mice was significantly greater than both *Fbn*^C1039G/+^ (0.09 ± 0.02 vs. 0.06 ± 0.02 mm/4 wk; *P* < 0.05; Fig. 2*C*) and *βarr2*^−/−^ (0.09 ± 0.02 vs. 0.02 ± 0.01 mm/4 wk; *P* < 0.05; Fig. 2*C*) mice.

In *Fbn*^C1039G/+^ mice, the rate of aortic dilation slowed over time from 0.11 ± 0.01 mm/4 wk from age 8 to 16 wk to 0.06 ± 0.02 mm/4 wk after 16 wk of age (*P* < 0.01; Fig. 2*D*). This finding is consistent with previous work, demonstrating a gradual decrease in the rate of aortic dilation in *Fbn*^C1039G/+^ mice (18). In contrast, the rate of aortic dilation in *Fbn*^C1039G/+^/*βarr2*^−/−^ increased over time from 0.03 ± 0.01 mm/4 wk from age 8 to 16 wk to 0.09 ± 0.02 mm/4 wk after 16 wk of age (*P* < 0.05; Fig. 2*D*). We observed no significant difference in aortic root diameter between wild-type and *βarr2*^−/−^ mice at 8 wk of age, as measured by repeated-measures, two-way ANOVA with post hoc Bonferroni testing (Fig. 2*F*).

#### Proaneurysmal gene and protein expression in aortic tissue is reduced in Fbn^C1039G/+^/βarr2^−/−^ mice compared with Fbn^C1039G/+^ mice.

**Fig. 3.**
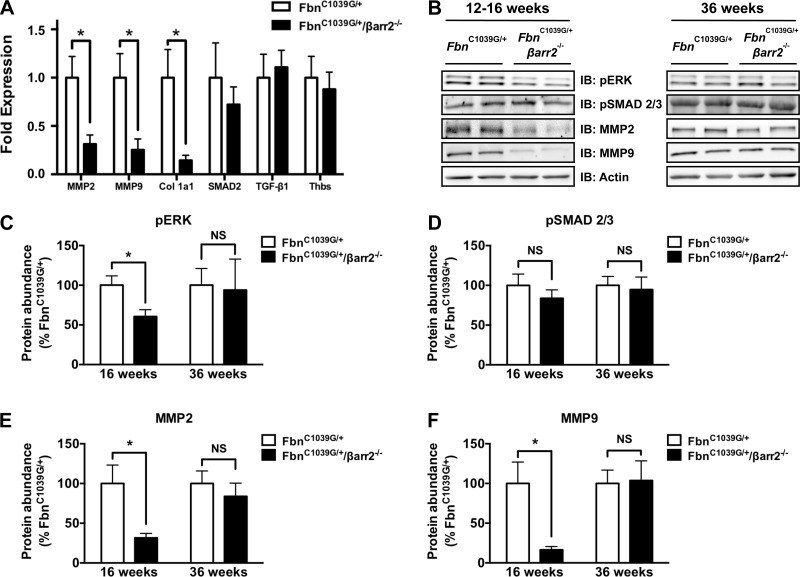
Matrix metalloproteinase (MMP)-2 and -9 gene and protein expression is reduced in TAA tissue of *Fbn*^C1039G/+^/*βarr2*^−/−^ mice relative to *Fbn*^C1039G/+^ mice. *A*: gene expression of MMP-2 and -9 in proximal ascending aortic tissue is decreased in *Fbn*^C1039G/+^/*βarr2*^−/−^ mice relative to *Fbn*^C1039G/+^ mice at 12–16 wk of age. In addition, expression of collagen 1a1 (Col 1a1) is decreased in proximal ascending aortic tissue from *Fbn*^C1039G/+^/*βarr2*^−/−^ mice compared with *Fbn*^C1039G/+^ mice, whereas no differences are observed in the expression levels of SMAD2, transforming growth factor (TGF)-β1, or thrombospondin-1 (Thbs). *B*: representative Western blots of TAA tissue of 2 *Fbn*^C1039G/+^ and 2 *Fbn*^C1039G/+^/*βarr2*^−/−^ mice between ages 12 and 16 wk (*left*) and 36 wk of age (*right*). *C*: ERK phosphorylation (p) is reduced in *Fbn*^C1039G/+^/*βarr2*^−/−^ mice relative to *Fbn*^C1039G/+^ mice between 12 and 16 wk of age, whereas no difference is observed between genotypes at 36 wk of age. NS, nonsignificant. *D*: no difference is observed in the level of pSMAD2/3 between genotypes at early or late time points. MMP-2 (*E*) and MMP-9 (*F*) protein expression is decreased at early time points in *Fbn*^C1039G/+^/*βarr2*^−/−^ mice relative to *Fbn*^C1039G/+^ mice, whereas no difference in expression levels is observed in TAA tissue of animals at 36 wk of age. For quantitative RT-PCR (qRT-PCR) experiments: *Fbn*^C1039G/+^, *n* = 14, and *Fbn*^C1039G/+^/*βarr2*^−/−^, *n* = 10; for Western analysis: *Fbn*^C1039G/+^, *n* = 8, and *Fbn*^C1039G/+^/*βarr2*^−/−^, *n* = 7; *P < 0.05.

Multiple proaneurysmal genes and proteins, such as MMP-2 and -9, are upregulated in aneurysmal aortic tissue in murine MFS models (8, 36, 37), as well as human MFS patient samples (32). In addition, AT_1a_R-mediated upregulation of these genes in the aorta has been suggested to be βarr2 dependent (33). Therefore, we assessed the gene expression of proaneurysmal signaling mediators in aneurysmal aortic tissue of mice, aged 12–16 wk, a time point where the largest differences in rate of dilation and aortic root diameter were observed between *Fbn*^C1039G/+^ and *Fbn*^C1039G/+^/*βarr2*^−/−^ mice. In particular, gene expression of both MMP-2 (0.31 ± 0.09- vs. 1.0 ± 0.22-fold; *P* < 0.05) and MMP-9 (0.25 ± 0.11- vs. 1.0 ± 0.25-fold; *P* < 0.05) is reduced significantly in *Fbn*^C1039G/+^/*βarr2*^−/−^ mice compared with *Fbn*^C1039G/+^ mice (Fig. 3*A*). In addition, gene expression of col 1a1 is reduced significantly in *Fbn*^C1039G/+^/*βarr2*^−/−^ mice relative to *Fbn*^C1039G/+^ mice (0.16 ± 0.06- vs. 1.0 ± 0.29-fold; *P* < 0.05; Fig. 3*A*). No difference was observed in the expression of TGF-β1, SMAD2, or thrombospondin-1, an ANG-stimulated potentiator of TGF-β signaling (Fig. 3*A*).

We also performed Western blot analysis of TAA tissue of *Fbn*^C1039G/+^ and *Fbn*^C1039G/+^/*βarr2*^−/−^ mice, aged 12–16 wk. pERK is reduced by ∼40% in *Fbn*^C1039G/+^/*βarr2*^−/−^ mice compared with *Fbn*^C1039G/+^ mice (60.6 ± 8.7% vs. 100 ± 11.6%; *P* < 0.05; Fig. 3, *B* and *C*). Notably, pSMAD2/3, a surrogate marker of canonical TGF-β signaling, is not significantly different between *Fbn*^C1039G/+^ and *Fbn*^C1039G/+^/*βarr2*^−/−^ mice (Fig. 3, *B* and *D*). MMP-2 and -9 protein expression is reduced by 69% (31.5 ± 5.7% vs. 100 ± 23.2%; *P* < 0.05) and 84% (16.4 ± 4.2% vs. 100 ± 26.9%; *P* < 0.05), respectively, in *Fbn*^C1039G/+^/*βarr2*^−/−^ mice compared with *Fbn*^C1039G/+^ mice (Fig. 3, *B*, *E*, and *F*).

We performed Western blot analysis of TAA tissue at 36 wk of age to assess the level of MMP-2 and -9, pERK1/2, and pSMAD2/3 at a time point where aortic size was similar between the two genotypes. Interestingly, no significant differences were observed in the expression levels of MMP-2 or -9 nor were differences observed in the levels of pERK1/2 or pSMAD2/3 at this later time point (Fig. 3, *B–F*).

#### ANG stimulates increased MMP-2 and -9 gene and protein expression in primary aortic root VSMCs.

**Fig. 4.**
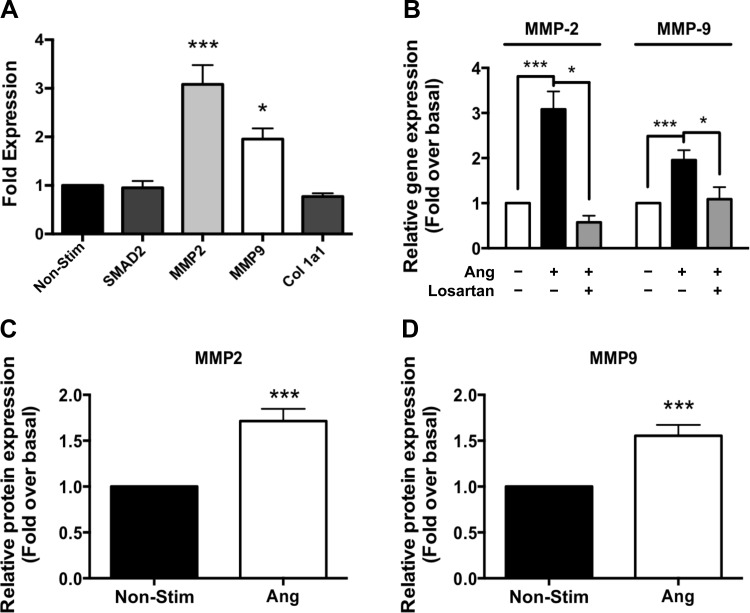
ANG-stimulated MMP-2 and -9 gene and protein expression is mediated via the ANG type 1a receptor (AT_1a_R) in primary aortic root vascular smooth muscle cells (VSMCs). *A*: by qRT-PCR analysis, ANG stimulates a 3.1-fold increase in MMP-2 gene transcription and a 2.0-fold increase in MMP-9 gene transcription. ANG stimulated; no significant increase is seen in SMAD2 or col 1a1 expression with ANG stimulation. *B*: MMP-2 and -9 gene expression is inhibited by blockade of the AT_1a_R with losartan. ANG stimulates a 1.7- and 1.6-fold increase in MMP-2 (*C*) and MMP-9 (*D*) protein expression. *P < 0.05 vs. nonstimulated (Non-Stim) unless other comparison noted; ***P < 0.001.

VSMC-derived MMP-2 and -9 have been identified previously to be upregulated in aortic samples from MFS patients, as well as in aortic samples in a murine model of MFS (5, 29). Work from our lab (7) has previously demonstrated that βarr2 does not interact with or augment signaling via the type I or II TGF-β receptors upon TGF-β stimulation. Therefore, we hypothesized that βarr2 might exert its effects on TAA formation in MFS via regulation of AT_1a_R signaling. To assess the direct effect of AT_1a_R-mediated signaling on the expression of the proaneurysmal genes MMP-2 and -9, we performed qRT-PCR on primary aortic VSMCs stimulated with ANG for 24 h. ANG stimulates a 3.1-fold increase in MMP-2 gene expression (*P* < 0.001) and a 2.0-fold increase in MMP-9 gene expression (*P* < 0.05; Fig. 4, *A* and *B*), with no significant stimulation of SMAD2 or col 1a1 gene expression in response to ANG treatment (Fig. 4*A*). ANG-stimulated increases in MMP-2 (*P* < 0.01) and MMP-9 (*P* < 0.05) gene expression are inhibited by blockade of the AT_1a_R with losartan (Fig. 4*B*). By Western blot analysis, we found that ANG stimulates a 1.7-fold increase in MMP-2 protein expression (*P* < 0.005; Fig. 4*C*) and a 1.6-fold increase in MMP-9 protein expression (*P* < 0.001; Fig. 4*D*) after 24 h of stimulation.

#### ANG-stimulated upregulation of MMP-2 and -9 gene and protein expression requires βarr2.

**Fig. 5.**
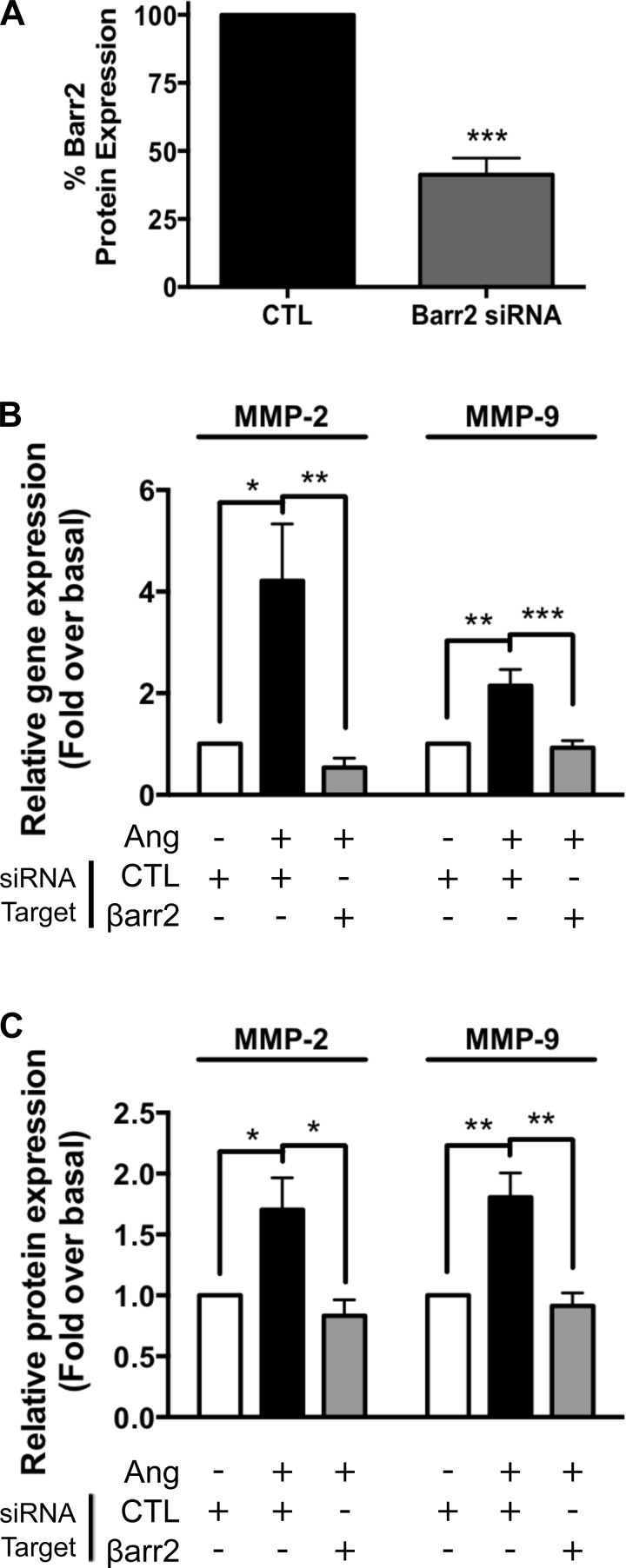
Angiotensin-stimulated MMP-2 and -9 expression is mediated by βarr2 in primary aortic root VSMCs. *A*: average βarr2 protein expression knockdown achieved was 62.3 ± 5.4%; *P* < 0.0001 vs. control (CTL)-transfected cells. *B*: ANG-stimulated MMP-2 and -9 gene expression is completely inhibited by small interfering RNA (siRNA) silencing of βarr2 expression. *C*: ANG-stimulated MMP-2 and -9 protein expression is completely inhibited by siRNA silencing of βarr2 expression. *P < 0.05, **P < 0.01, ***P < 0.001 vs. nonstimulated or CTL condition unless otherwise demarcated; *n* = 5 independent cell lines for experiments with siRNA targeting βarr2.

As noted, we observed decreased gene and protein expression of MMP-2 and -9 in *Fbn*^C1039G/+^/*βarr2*^−/−^ mice relative to *Fbn*^C1039G/+^ mice (Fig. 3). To assess directly the potential role of βarr2 in mediating this proaneurysmal signaling, we transfected primary aortic root VSMC with siRNA targeting βarr2 and assessed the ability of ANG to stimulate profibrotic gene expression. By Western blot analysis, the average reduction in βarr2 protein expression achieved with siRNA treatment targeting βarr2 was 62.3 ± 5.4%; *P* < 0.0001 vs. control-transfected cells (Fig. 5*A*). By qRT-PCR, we observed that ANG-stimulated increases in MMP-2 (4.2 ± 1.1- vs. 0.5 ± 0.2-fold; *P* < 0.01) and MMP-9 (2.1 ± 0.3- vs. 0.9 ± 0.1-fold; *P* < 0.001) gene transcription are completely abolished in the presence of siRNA targeting βarr2 (Fig. 5*B*). By Western blot analysis, we observed that ANG-stimulated MMP-2 (1.7 ± 0.3- vs. 0.8 ± 0.1-fold; *P* < 0.05) and MMP-9 (1.8 ± 0.2- vs. 0.9 ± 0.1-fold; *P* < 0.01) protein production is inhibited in the presence of siRNA targeting βarr2 (Fig. 5*C*).

#### ANG-stimulated upregulation of MMP-2 and -9 gene and protein expression is dependent on ERK1/2 activation.

**Fig. 6.**
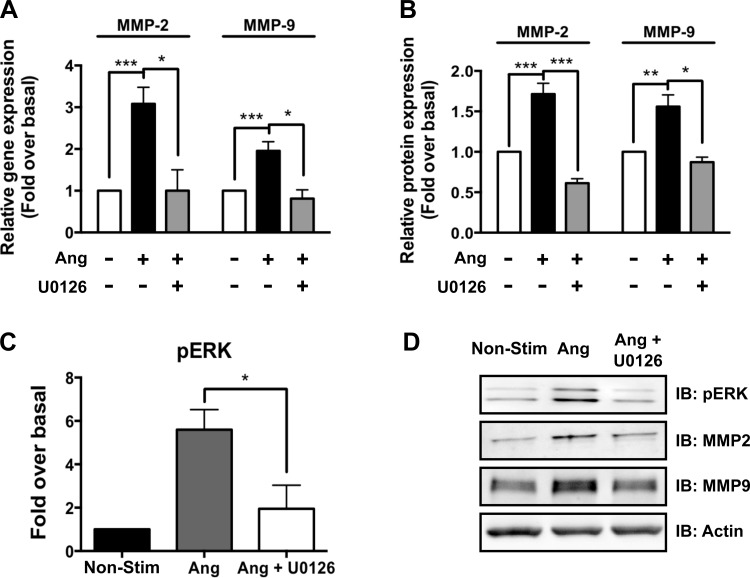
ANG-stimulated MMP-2 and -9 gene and protein expression is dependent on ERK1/2 activation in primary aortic root VSMCs. *A*: ANG-stimulated gene expression of MMP-2 and -9 is blocked by MEK1/2 inhibition with U0126. *B*: ANG-stimulated protein expression of MMP-2 and -9 is also blocked by MEK1/2 inhibition with U0126. *C*: ANG-stimulated pERK1/2 is completely blocked by inhibition of MEK1/2 with U0126. *D*: Western blot of the effect of MEK1/2 inhibition with U0126 on ANG-stimulated MMP-2 and -9 expression representative of 5–6 individual experiments. *P = <0.05, **P < 0.01, ***P < 0.001.

Consistent with our data, demonstrating reduced ERK1/2 activation in aneurysmal tissue from *Fbn*^C1039G/+^/*βarr2*^−/−^ mice compared with *Fbn*^C1039G/+^ mice (Fig. 3*C*), blockade of ERK1/2 with the MEK1/2 inhibitor U0126 completely abolishes ANG-stimulated increases in MMP-2 (3.1 ± 0.4- vs. 1.0 ± 0.5-fold; *P* < 0.05) and MMP-9 (2.0 ± 0.2- vs. 0.8 ± 0.2-fold; *P* < 0.05) gene expression (Fig. 6*A*). ANG-stimulated protein expression of MMP-2 (1.7 ± 0.4- vs. 0.6 ± 0.1-fold; *P* < 0.001) and MMP-9 (1.6 ± 0.4- vs. 0.9 ± 0.03-fold; *P* < 0.01) is also completely abolished by inhibition of MEK1/2 with U0126 (Fig. 6*B*). Notably, blockade of ANG-stimulated ERK1/2 activation was markedly reduced with blockade of ERK1/2 with U0126 (5.6 ± 0.9- vs. 2.0 ± 1.0-fold; *P* < 0.05; Fig. 6*C*).

#### EGFR transactivation contributes to MMP-2 and -9 gene and protein expression as well as TAA formation in MFS.

**Fig. 7.**
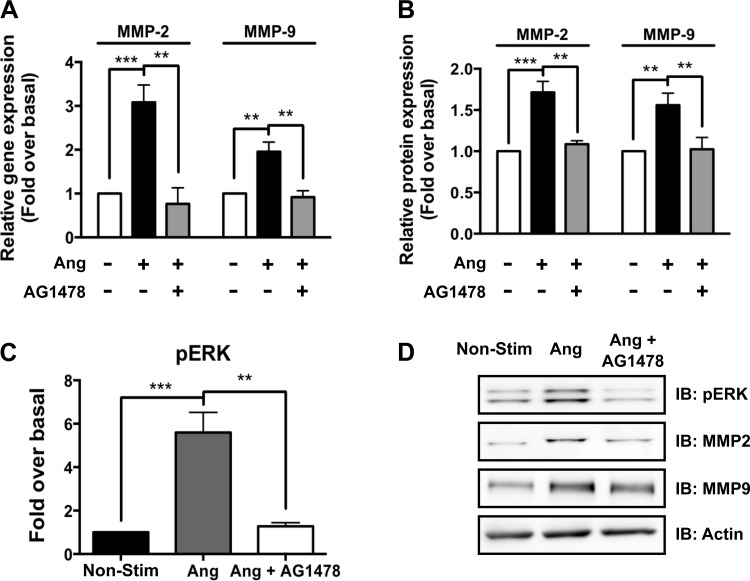
ANG-stimulated MMP-2 and -9 gene and protein expression is dependent on the EGF receptor (EGFR) in primary aortic root VSMCs. *A*: ANG-stimulated gene expression of MMP-2 and -9 is blocked by EGFR inhibition with the EGFR inhibitor AG1478. *B*: ANG-stimulated protein expression of MMP-2 and -9 is also blocked by EGFR inhibition with the EGFR inhibitor AG1478. *C*: ANG-stimulated pERK1/2 at 15 min is completely blocked by inhibition of EGFR with AG1478. *D*: Western blot of the effect of EGFR inhibition with AG1478 of ANG-stimulated MMP-2 and -9 production representative of 4–8 individual experiments. **P < 0.01, ***P < 0.001.

Previous work has demonstrated that AT_1a_R-dependent, βarr2-mediated ERK1/2 activation requires transactivation of the EGFR (21). Therefore, we examined whether EGFR transactivation was required for AT_1a_R-dependent MMP-2 and -9 expression. By qRT-PCR, we found that inhibition of EGFR transactivation with AG1478 inhibits ANG-stimulated increases in MMP-2 (3.1 ± 0.4- vs. 0.8 ± 0.4-fold; *P* < 0.01) and MMP-9 (2.0 ± 0.2- vs. 0.9 ± 0.1-fold; *P* < 0.05) gene expression (Fig. 7*A*). We also found that ANG-stimulated MMP-2 (1.7 ± 0.4- vs. 1.1 ± 0.04-fold; *P* < 0.05) and MMP-9 (1.6 ± 0.4- vs. 1.0 ± 0.1-fold; *P* < 0.01) protein production was inhibited by blockade of EGFR transactivation with AG1478 (Fig. 6*B*). ANG-stimulated ERK1/2 activation is reduced significantly in the presence of AG1478, confirming the requirement of EGFR transactivation in ANG-stimulated ERK1/2 activation (Fig. 7*C*).

**Fig. 8.**
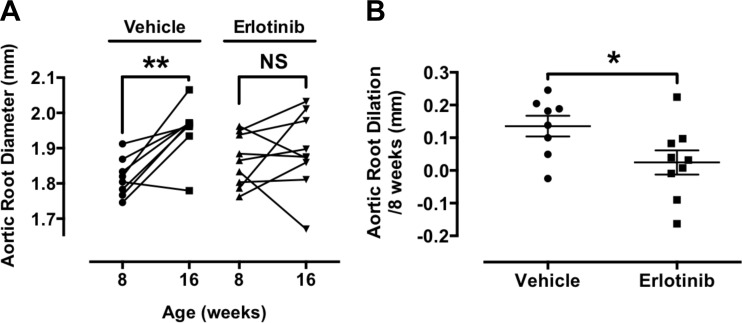
Pharmacologic inhibition of the EGFR blocks thoracic aortic dilation in a murine model of MFS. *A*: *Fbn1*^C1039G/+^ mice were treated with subcutaneous infusion of either vehicle (*left*) or erlotinib (*right*) for 8 wk, beginning at 8 wk of age. Aortic root diameter was followed by transthoracic echocardiography at 8 and 16 wk of age. No significant aortic dilation was observed in *Fbn1*^C1039G/+^ mice treated with erlotinib, whereas *Fbn1*^C1039G/+^ mice treated with placebo displayed significant aortic dilation. NS, nonsignificant. *B*: the rate of change of aortic root diameter was significantly less in *Fbn1*^C1039G/+^ mice treated with erlotinib (*right*) compared with vehicle-treated mice (*left*). *P = <0.05, **P < 0.01 by *t*-test.

To evaluate the role of EGFR activation in vivo, we treated *Fbn*^C1039G/+^ mice with subcutaneous infusion of the EGFR inhibitor erlotinib (5 mg/ml) for 8 wk and followed aortic root diameter by transthoracic echocardiography. We observed no significant aortic dilation in erlotinib-treated mice (1.87 ± 0.02 vs. 1.89 ± 0.04 mm; *P* = NS), whereas vehicle-treated mice demonstrated significant aortic dilation over 8 wk (1.82 ± 0.02 vs. 1.95 ± 0.03 mm; *P* < 0.01; Fig. 8*A*). Vehicle-treated *Fbn*^C1039G/+^ mice displayed an absolute increase of 0.13 ± 0.03 mm compared with 0.02 ± 0.04 in erlotinib-treated mice (*P* < 0.05; Fig. 8*B*).

#### ANG-stimulated upregulation of MMP-2 and -9 gene and protein expression is independent of TGF-β.

**Fig. 9.**
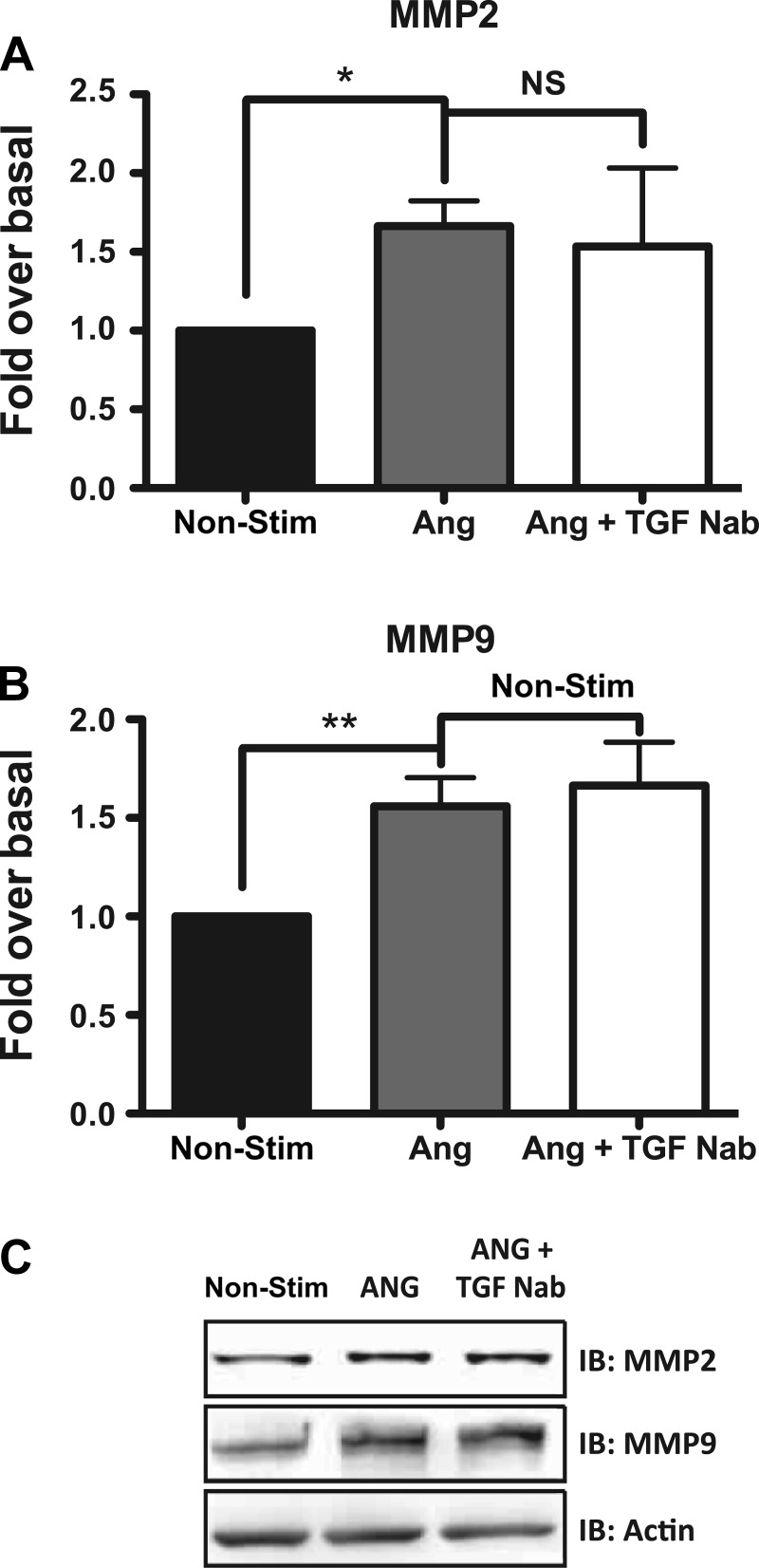
ANG-stimulated MMP-2 and -9 protein expression is independent of TGF-β in primary aortic root VSMCs. ANG-stimulated MMP-2 (*A*) and MMP-9 (*B*) protein expression is unaffected by blockade of TGF-β with a pan-specific neutralizing antibody (Nab) against TGF-β1, -2, and -3. *C*: representative Western blot of the effect of blockade of TGF-β signaling with a neutralizing antibody on ANG-stimulated MMP-2 and -9 production. Blot is representative of 4 independent experiments. *P < 0.05, **P < 0.001; *n* = 4.

As noted previously, we observed no change in the activation of SMAD2/3 in thoracic aortic tissue from *Fbn*^C1039G/+^/*βarr2*^−/−^ mice compared with *Fbn*^C1039G/+^ mice (Fig. 3*D*). By qRT-PCR, the expression levels of TGF-β1, SMAD2, and thrombospondin-1 were also unchanged in the TAA tissue of *Fbn*^C1039G/+^/*βarr2*^−/−^ mice compared with *Fbn*^C1039G/+^ mice (Fig. 3*A*), suggesting that the role of βarr2 in mediating TAA formation in MFS is independent of TGF-β. We also assessed whether TGF-β was indirectly involved in βarr2-mediated proaneurysmal signaling in primary aortic root VSMCs. ANG-stimulated protein expression of MMP-2 (1.7 ± 0.4- vs. 1.6 ± 0.7-fold; *P* = NS; Fig. 8*A*) and MMP-9 (1.6 ± 0.4- vs. 1.7 ± 0.2-fold; *P* = nonstimulated; Fig. 9*B*) is unaffected by blockade of TGF-β signaling with a pan-specific neutralizing antibody against TGF-β1, -2, and -3, indicating that this signaling cascade is independent of TGF-β.

## DISCUSSION

Here, we demonstrate that βarr2 contributes to TAA formation in a murine model of MFS. In particular, we observed that *Fbn*^C1039G/+^/*βarr2*^−/−^ mice display delayed aortic root dilation relative to *Fbn*^C1039G/+^ mice. We found that gene and protein expression of MMP-2 and -9 is markedly reduced in TAA tissue obtained between 12 and 16 wk of age from *Fbn*^C1039G/+^/*βarr2*^−/−^ mice relative to *Fbn*^C1039G/+^. We hypothesized that βarr2 mediates its affects on TAA development via regulation of AT_1a_R signaling in VSMCs. We show that ANG-induced MMP-2 and -9 expression requires βarr2 in primary aortic root VSMCs. This pathway is mediated by both ERK1/2 activation as well as EGFR transactivation. We also observed that levels of MMP-2 and -9 and pERK1/2 varied over time in *Fbn*^C1039G/+^/*βarr2*^−/−^ mice relative to *Fbn*^C1039G/+^ mice. This finding suggests that the signaling pathways that contribute to aortic root dilation in MFS may vary over time. This idea is supported by previous work demonstrating that microRNA-29b contributes to aortic root dilation only at early time points in a murine model of MFS (27). The recognition of the influence of this signaling pathway identifies a contributory signaling mechanism to TAA pathogenesis in MFS that may potentially serve as a target for pharmacologic inhibition.

Our study is corroborated by recent work demonstrating that AT_1a_R-dependent, βarr2-mediated signaling contributes to abdominal aortic aneurysm (AAA) formation in an ANG-infusion model of AAA (33). Although this mouse model is distinct from the model evaluated in this study, the authors showed that ANG-induced AAA formation and MMP-2 and -9 expression in the abdominal aorta are attenuated by genetic *βarr2*^−/−^. We have identified a similar βarr2-dependent signaling mechanism present in the thoracic aorta. In particular, we demonstrate that βarr2-dependent MMP-2 and -9 expression requires both ERK1/2 and EGFR activation. We propose that this pathway contributes to TAA formation via regulation of the expression of proaneurysmal mediators MMP-2 and -9. Beyond TAA development, AT_1a_R-mediated, βarr2-dependent signaling in MFS has also been shown to contribute to the development of a primary dilated cardiomyopathy in a murine model of MFS (10), providing further corroborative evidence for a deleterious role of AT_1a_R-mediated, βarr2-dependent signaling in MFS.

**Fig. 10.**
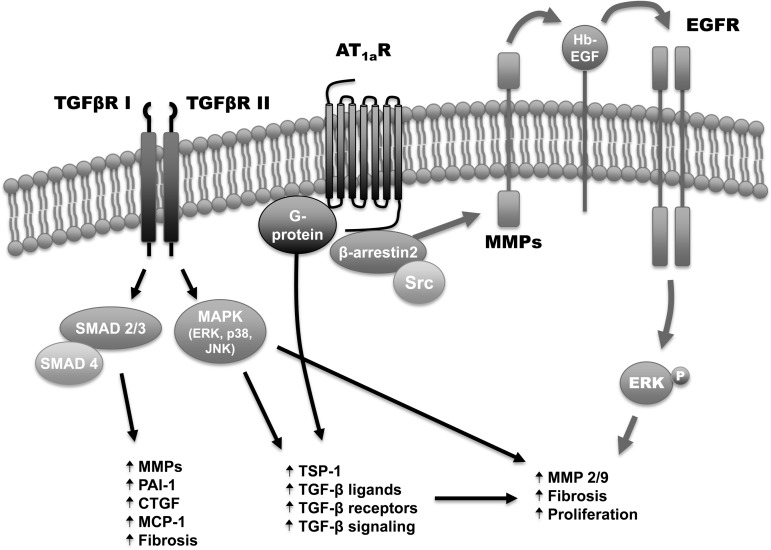
Proposed role of noncanonical AT_1a_R signaling in TAA development in MFS. Angiotensin stimulates both canonical G protein-dependent signaling as well as noncanonical, βarr2-dependent signaling via the AT_1a_R in the thoracic aorta. βarr2-dependent signaling mediates transactivation of the EGFR via MMP-dependent cleavage of heparin-bound EGF (Hb-EGF). This, in turn, results in pERK1/2 with a resultant increase in MMP-2 and -9 gene and protein production, as well as increased fibrosis and VSMC proliferation. TGFβR I/II, types I and II TGF-β receptor; P, phosphate; PAI-1, plasminogen activator inhibitor 1; CTGF, connective tissue growth factor; MCP-1, monocyte chemoattractant protein-1; TSP-1, thrombospondin-1.

Habashi et al. (18) have previously proposed a mechanism by which ANG contributes to TAA formation in MFS through three distinct pathways: *1*) via upregulation of TGF-β ligands and receptors; *2*) by upregulation of TGF-β signaling, directly as well as through upregulation of thrombospondin-1, a potentiator of TGF-β signaling; and *3*) by directly stimulating VSMC proliferation, decreasing apoptosis, increasing profibrotic signaling, and upregulating MMP-2 and -9 (25). We propose that this third mechanism of ANG contribution to TAA development in MFS is βarr2 dependent (Fig. 10). Previous work from our lab (21, 22) has demonstrated that βarr2 mediates ANG-induced VSMC proliferation, as well as ANG-induced antiapoptotic signaling through regulation of Bcl-2-associated death promoter phosphorylation (1). In addition, recent work suggests that βarr2 mediates proaneurysmal signaling and MMP-2 and -9 expression in abdominal aortic tissue (33).

Importantly, this work underscores the significance of ERK-dependent signaling in TAA development in MFS. ERK-dependent signaling has been demonstrated previously to contribute to aortic dilation in MFS (18, 20). ANG stimulates ERK1/2 activation via the AT_1a_R and both G_q_ proteins as well as βarr2. ERK activated via these different transducers is both spatially and temporally distinct (3) with unique functional outcomes (22, 34). Whereas βarr2-dependent ERK activation appears to lead to TGF-β-independent, proaneurysmal signaling, ANG-stimulated activation of TGF-β signaling has been reported previously to involve G_q_ proteins (35). Interestingly, G protein- and βarr2-dependent ERK1/2 activation has been shown to require EGFR transactivation in VSMC (21), suggesting the EGFR could serve as a mediator of AT_1a_R-mediated pathogenic signaling in MFS. This hypothesis is supported by our preliminary work demonstrating a reduction in aortic dilation in *Fbn*^C1039G/+^ mice treated with the EGFR inhibitor erlotinib (Fig. 7).

There are several limitations of this study. First, whereas we have identified a role for βarr2 in the pathogenesis of TAA in MFS, the complex interplay of intracellular signaling events and ECM deficiencies in MFS makes in vitro recapitulation in cell culture systems of in vivo signaling events difficult. We demonstrate that pERK and MMP-2 and -9 are reduced significantly in aneurysmal tissue from *Fbn*^C1039G/+^/*βarr2*^−/−^ mice relative to *Fbn*^C1039G/+^ mice. ERK has been established previously as a key mediator of TAA formation in this model of MFS (18, 20). We used primary aortic root VSMCs and demonstrated that βarr2 mediates ANG-stimulated ERK activation and MMP-2 and -9 production in VSMCs from the aortic root. As noted previously, MMP-2 and -9 production has been shown to be elevated in VSMCs from patients with MFS (29), as well as in a murine model of MFS (5). Therefore, we believe that our in vitro work represents a novel and relevant finding with regard to the signaling pathways involved in MMP-2 and -9 production in the aortic root. Nevertheless, it is possible that βarr2-mediated ERK activation and MMP-2 and -9 production in other cell types, such as endothelial cells, adventitial fibroblasts, or inflammatory cells, could contribute to TAA formation in MFS. This is currently the subject of ongoing investigation in our laboratory.

Additionally, it should be noted that our in vitro signaling work was performed in VSMCs harvested from rats as opposed to mice. We chose to use VSMCs harvested from rats due to a priori concerns regarding the number of animals and cells necessary for study. Because we planned to perform in vitro assays in VSMCs harvested as close to the aortic root as possible, we expected that VSMC yields from mice would be far too low for our needs and require a large number of animals (100–200). Therefore, we had significant ethical and technical concerns regarding the use of murine VSMCs for this portion of our study. Notably, previous work in our lab (21) and others' (16) has demonstrated that AT_1a_R-mediated ERK activation involves similar pathways in both murine and rat aortic VSMC, suggesting that rat VSMCs could serve as a viable model system in which to recapitulate the signaling pathways of interest in our study.

Second, βarr2-dependent effects appear to predominate at early time points in this murine model of MFS. This occurs concomitantly with a decrease in the levels of pERK and MMP-2 and -9 expression in the aortic root. Interestingly, over time, both aortic root diameter and MMP-2 and -9 expression in *Fbn*^C1039G/+^/*βarr2*^−/−^ mice return to levels similar to *Fbn*^C1039G/+^ mice. The precise mechanisms underlying this phenomenon are unclear; however, it suggests that the key contributors to TAA formation in MFS may differ over time and result in disparate rates of dilation. Again, this hypothesis is supported by previous work demonstrating a role for microRNA-29b solely in early TAA development in a murine model of MFS (27). This time-dependent pattern of βarr2 effect is also the subject of ongoing investigation in our laboratory.

In conclusion, this work identifies a previously unrecognized signaling cascade that contributes to TAA formation in MFS. It also emphasizes the idea that TAA development in MFS is the end result of an interconnected network of signaling involving multiple receptor types and signaling mediators (Fig. 10), as well as ECM-dependent processes (18, 19, 25). In addition, it identifies βarr2 as a potential mediator of TAA formation and broadens our understanding of the complex signaling pathways involved in aortic aneurysmal disease. This may, in turn, lead to the development of more targeted therapeutics for the prevention and/or treatment of TAAs in patients with MFS. This work should be viewed as hypothesis generating, in that it identifies multiple, potential targets of inhibition, such as AT_1a_R, TGF-β, MMP-2 and -9, ERK1/2, EGFR, or βarr2. This is supported by previous work demonstrating the benefit of blockade of AT_1a_R (19), TGF-β (19), MMP-2 and -9 (9, 36), and ERK1/2 (20) in murine models of MFS, as well as our own preliminary data demonstrating reduced aortic dilation in this model of MFS with pharmacologic inhibition of the EGFR (Fig. 8). Of particular interest is the recognition that pharmacologic agents currently in clinical use for other indications, such as inhibitors of EGFR and MMPs, may have use in the prevention or treatment of TAA formation in MFS.

## GRANTS

Support for this work was provided by the National Heart, Lung, and Blood Institute Grants HL16037 and HL70631 to R. J. Lefkowitz; HL56687 and HL75443 to H. A. Rockman; and Institutional Grant T32 HL007101 to J. W. Wisler. R. J. Lefkowitz is an investigator with the Howard Hughes Medical Institute.

## DISCLOSURES

R. J. Lefkowitz and H. A. Rockman are scientific cofounders of Trevena, a company developing biased G protein-coupled receptor ligands.

## AUTHOR CONTRIBUTIONS

Author contributions: J.W.W., J.K., and H.A.R. conception and design of research; J.W.W., E.M.H., M.R., and L.M. performed experiments; J.W.W. drafted manuscript; J.W.W., J.K., H.A.R., and R.J.L. edited and revised manuscript; J.W.W., H.A.R., and R.J.L. approved final version of manuscript; J.W.W. analyzed data; J.W.W., E.M.H., and R.J.L. interpreted results of experiments; J.W.W. prepared figures.
